# Improving diagnostic accuracy in assessing pulmonary edema on bedside chest radiographs using a standardized scoring approach

**DOI:** 10.1186/1471-2253-14-94

**Published:** 2014-10-18

**Authors:** Matthias Hammon, Peter Dankerl, Heinz Leonhard Voit-Höhne, Martin Sandmair, Ferdinand Josef Kammerer, Michael Uder, Rolf Janka

**Affiliations:** Department of Radiology, University Hospital Erlangen, Maximiliansplatz 1, 91054 Erlangen, Germany; Department of Radiology, Hospital Ansbach, Escherichstrasse 1, 91522 Ansbach, Germany

**Keywords:** Bedside chest radiograph, Pulmonary edema, Scoring system, Extravascular lung water (EVLW), Web-based application

## Abstract

**Background:**

To assess the value of a score-based system which allows standardized evaluation of pulmonary edema on bedside chest radiographs (CXRs) under routine clinical conditions.

**Methods:**

Seven experienced readers assessed bedside CXRs of ten patients with an extravascular lung water (EVLW)-value of ≤ 8 mL/kg (range: 4–8 mL/kg; indicates no pulmonary edema) and a series of ten patients with an EVLW-value of ≥ 15 mL/kg (range: 15–21 mL/kg; = indicates a pulmonary edema) with and without customized software which would permit a standardized assessment of the various indications of pulmonary edema. The software provides a score that identifies patients with and without pulmonary edema. EVLW-values were measured instantly after bedside CXR imaging using a pulse contour cardiac output (PiCCO) system and served as a reference standard. The patients were non-traumatic and not treated with diuretics or dobutamine during bedside CXR imaging and the PiCCO measurements. Mean sensitivity, specificity, positive and negative predictive value, the percentage of overall agreement and the free-marginal multirater kappa value was calculated for both the standard and the standardized score-based approach. The net reclassification index was calculated for each reader as well as for all readers.

**Results:**

Evaluation of bedside CXRs by means of the score-based approach took longer (23 ± 12 seconds versus 7 ± 3 seconds without the use of the software) but improved radiologists’ sensitivity (from 57 to 77%), specificity (from 90 to 100%) and the free-marginal multirater kappa value (from 0.34 to 0.68). The positive predictive value was raised from 85 to 100% and the negative predictive value from 68 to 81%. A net reclassification index of 0.3 (all readers) demonstrates an improvement in prediction performance gained by the score-based approach. The percentage of overall agreement was 67% with the standard approach and 84% with the software-based approach.

**Conclusions:**

The diagnostic accuracy of bedside CXRs to discriminate patients with elevated EVLW-values from those with a normal value can be improved with the use of a standardized score-based approach. The investigated system is freely available as a web-based application (accessible via: http://www.radiologie.uk-erlangen.de/aerzte-und-zuweiser/edema).

## Background

The treatment of critically ill ventilated patients in an intensive care unit (ICU) is a demanding task for all involved. One important task is to assess the hemodynamic status and the presence of an increased intra- and extravascular volume which could potentially cause a pulmonary edema, which might negatively affect a patient’s ventilation and condition. Various methods are, therefore, available which enable the intra- and extravascular volume status and pulmonary edema to be assessed. There are non-invasive techniques, such as the evaluation of various indicators on chest radiographs (CXRs) or sonographic analysis of the presence of Kerley lines, which depict thickened interlobular septa [1]. Invasive techniques such as the indicator dilution method which measures the extravascular lung water (EVLW) using a pulse contour cardiac output (PiCCO) system (Pulsion Medical Systems, Munich, Germany) seem to be reliable for evaluation of pulmonary edema in patients with indirect lung injury [2], but they are expensive and there is a risk of complications developing. Under routine clinical conditions, the diagnosis of an altered hemodynamic status and the presence, and severity of, or changes to a pulmonary edema is commonly based on the evaluation of characteristic indicators on CXRs. An accurate discrimination of patients with and without pulmonary edema during evaluation of frequent bedside CXRs remains clinically important and valuable for the practicing physician in the ICU. In addition, the interpretation of bedside CXRs is known to be challenging [3–5], and mechanical ventilation may affect the appearance of the characteristic radiographic indicators [6]. It has been shown that the correlation between evaluation results and the actual EVLW-value can be weak when the customary reporting approach is chosen [7, 8]. New strategies are, therefore, needed to provide a more reliable evaluation of bedside CXRs. It has been shown, that, in the case of radiological evaluations, a scoring-based approach which allows a standardized analysis of various radiographic indicators can be beneficial in a variety of tasks [9–12]. We developed and assessed customized scoring software, implemented as a web-based application, which facilitates a standardized assessment of the presence of pulmonary edema. It is based on the systematic evaluation of characteristic radiological indications of pulmonary edema on bedside CXRs. Classification of the different indications leads to a total score that distinguishes patients with pulmonary edema from patients without pulmonary edema. In contrast to bedside CXRs a significant effort has been spent in order to improve evaluation of pulmonary edema which is detected on upright CXRs. One promising approach is the accurate assessment of the vascular pedicle. This makes it particularly easy to distinguish between pulmonary edema due to congestive heart failure, renal failure and acute lung injury [13–16]. To our knowledge no standardized scoring approach to assess pulmonary edema on bedside CXRs was previously correlated with a reference standard such as the EVLW-value.

Therefore, the purpose of this study was to investigate whether the proposed standardized score-based assessment of bedside CXRs increases diagnostic accuracy for radiologists when distinguishing between normal and elevated EVLW-values.

## Methods

This single-centre investigation was approved by the institutional review board of the University of Erlangen and all procedures were in accordance with the Helsinki Declaration. The need for informed consent was waived.

### Patient selection and pulse contour cardiac output (PiCCO) measurements

A number of possibly suitable patients was selected prospectively. We included non-traumatic, intubated and mechanically ventilated patients who were being treated in the ICU of the University Hospital Erlangen and who had a PiCCO catheter in place. PiCCO measurements of the EVLW [mL/kg] were taken to serve as a quantifiable reference standard of pulmonary edema. We included patients who had undergone bedside CXR imaging and where PiCCO measurements had been taken immediately afterwards. These were recorded by experienced intensive care nurses. Patients did not present direct lung injury and were not treated with diuretics or dobutamine while this was happening.

Although there is discussion on the use of PiCCO measurements, especially regarding the normal clinical range of EVLW as well as on the effect of the distribution of perfusion on EVLW values [17, 18], there is evidence that the EVLW-value is a sensitive marker of pulmonary edema [19–21]. Roch et al. provided evidence in an animal model that EVLW measurements are useful and reliable for evaluation of pulmonary edema in indirect lung injury, but produce misleading values in direct lung injury [2]. As all patients included in this study show indirect lung injury, we assume that the determined EVLW values are reliable. We defined an EVLW score of ≤ 8 mL/kg as no pulmonary edema, and a score of ≥ 15 mL/kg as pulmonary edema [8, 19, 22–27]. Patients presenting a borderline EVLW-value (9–14 mL/kg) and who, therefore, could not be reliably classified, were not included. A sequence of ten patients with an EVLW-value of ≤ 8 mL/kg and a series of ten patients with an EVLW-value of ≥ 15 mL/kg were included. In addition, patients were selected without prior knowledge of their clinical diagnosis.

### Radiographic technique

All patients underwent bedside chest radiography in the supine position (antero-posterior beam projection) with the use of a commercially available portable apparatus (Mobilett XP Digital, Siemens, Erlangen, Germany). Experienced radiology technologists performed the chest radiography (inspiratory view), using a standard technique. An ADC Compact system (Agfa HealthCare, Bonn, Germany) with compatible imaging plates (35/43) with a grid (70 lines, parallel, ratio 1:6) was used. The distance between X-ray tube and plate was 1.15 m.

### Scoring system

Customized scoring software, implemented as a web-based application, facilitates a standardized assessment of the presence of pulmonary edema. It is based on the systematic evaluation of characteristic radiological indications of pulmonary edema on bedside CXRs which was previously validated [1, 13, 28, 29].

**Figure 1:**
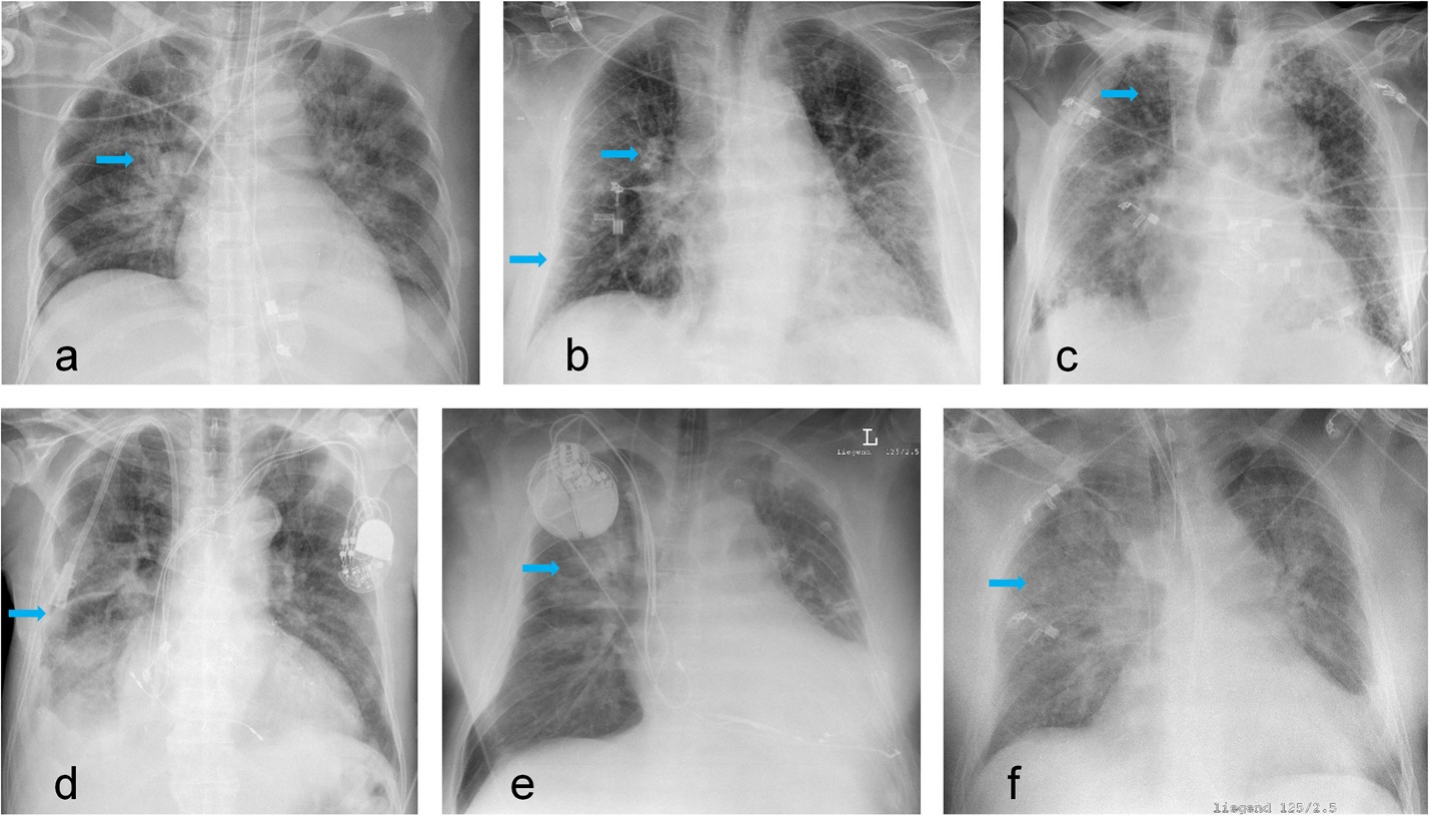
**Bedside chest radiographs showing different indications of pulmonary edema.** Enlarged/blurred hilar vessels **(a)**, Kerley lines (lower arrow) and peribronchial/perivascular cuffs (upper arrow) **(b)**, micronoduli **(c)** widening of interlobular fissure **(d)**, extensive perihilar haze **(e)** and diffuse increase in density **(f)**.

Each characteristic indication (a-g) has to be classified in a three point scale (b and c) as missing, moderate and severe or a four point scale (a, d-g) as missing, mild, moderate and severe: (a) hilar vessels enlarged (0/1/2/3 points), hilar vessels increased in density (0/2/4/6 points), hilar vessels blurred (0/3/6/9 points)), (b) Kerley B lines (0/4/-/8 points), (c) micronoduli (0/4/-/8 points), (d) widening of interlobular fissure (0/4/8/12 points), (e) peribronchial and perivascular cuffs (0/4/8/12 points), (f) extensive perihilar haze (0/5/10/15 points) and (g) diffuse increase in density (0/5/10/15 points). The software calculates the total score, and results > 15 are rated as pulmonary edema, whereas a score ≤ 15 is rated as no pulmonary edema. Detailed information of the radiological scoring of pulmonary edema is given in Figure 1 and Table 1.

**Table 1:** **Radiologic scoring of pulmonary edema on bedside chest radiographs**

Variables		Score		
		Mild	Moderate	Severe
Hilar vessels	Enlarged	1	2	3
	Increased in density	2	4	6
	Blurred	3	6	9
Kerley B lines		4	-	8
Micronoduli		4	-	8
Widening of interlobular fissure		4	8	12
Peribronchial and perivascular cuffs		4	8	12
Extensive perihilar haze		5	10	15
Diffuse increase in density		5	10	15

The score assigned to each variable depends on the severity of involvement (missing = 0 points, mild, moderate and severe). During the score-based approach a total score > 15 was rated as pulmonary edema, whereas a score ≤ 15 was rated as no pulmonary edema. The variables and scores were previously validated [1, 13, 28, 29].

### Image interpretation

**Figure 2:**
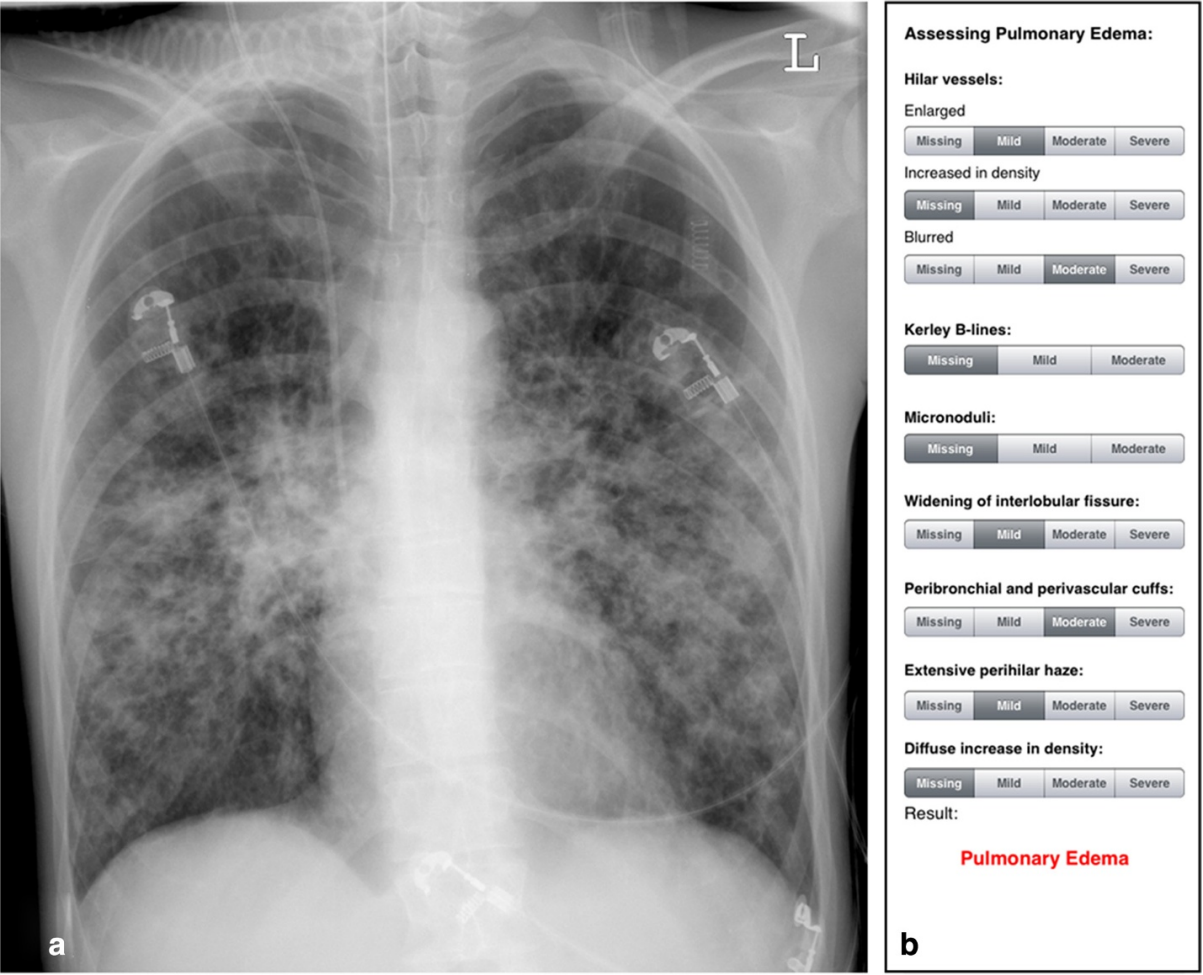
**Example bedside chest radiograph with indications of pulmonary edema. a)** Pulse contour cardiac output (PiCCO) measurements showed an extravascular lung water (EVLW)-value of 21 mL/kg, indicating pulmonary edema. **b)** Results of the score-based approach (total score = 24, indicates pulmonary edema).

**Figure 3:**
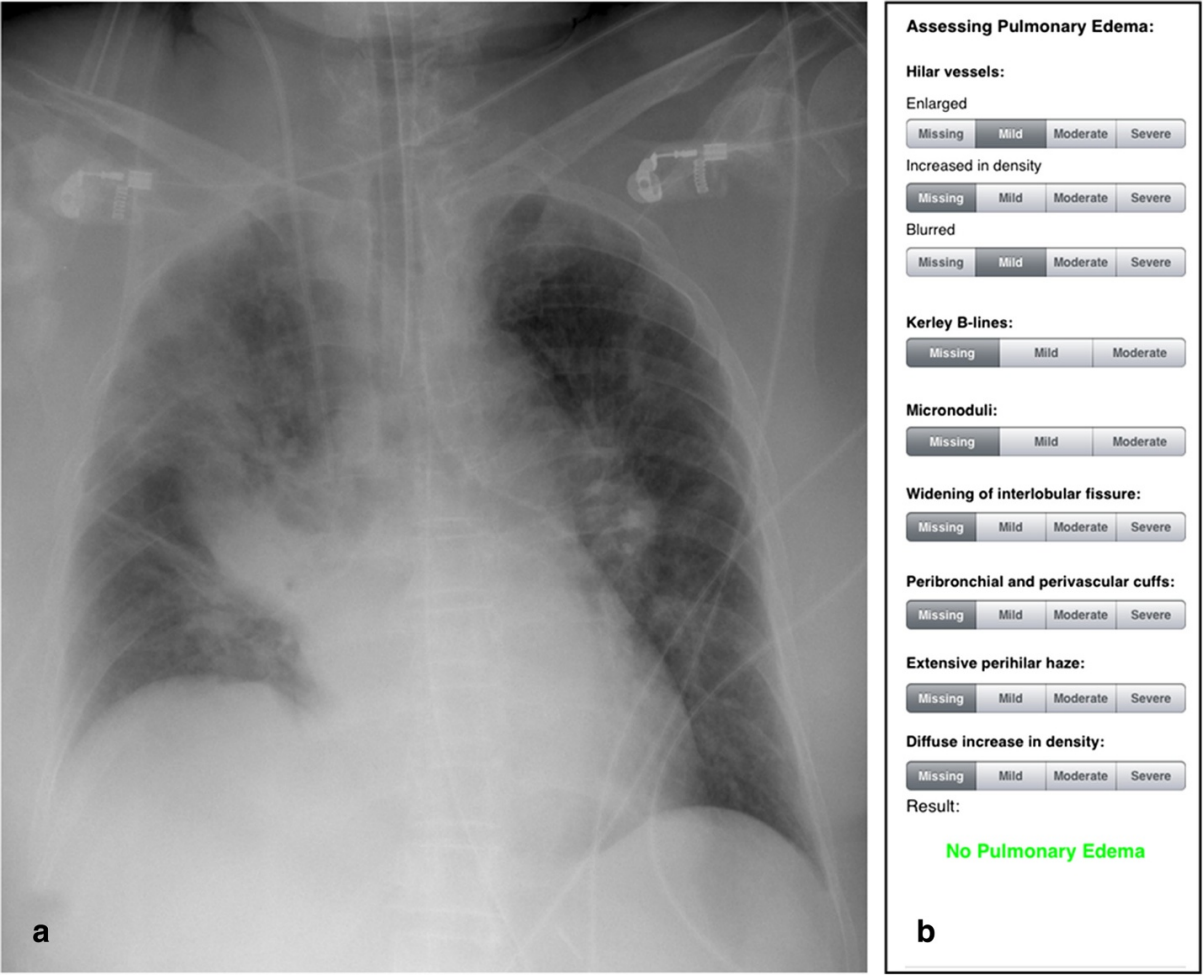
**Example bedside chest radiograph with no indications of pulmonary edema. a)** Pulse contour cardiac output (PiCCO) measurements showed an extravascular lung water (EVLW)-value of 7 mL/kg, indicating no pulmonary edema. **b)** Result of the score-based approach (total score = 4, indicates no pulmonary edema).

Images were labelled randomly by number, thus obscuring any reference to patient by name or age, and were stored and displayed by means of a commercially available picture achieving and communicating (PACS) system (Syngo Plaza, Siemens AG, Erlangen, Germany). We could not blind physicians concerning patient’s gender because it is usually apparent from the CXR. Afterwards, bedside CXRs were evaluated by seven radiologists (3–8 years of work experience) using a commercially available workstation. Each of them assessed the images independently without clinical information about the patients. The radiologists were not informed of the overall number of radiographs or the number of patients with normal or elevated EVLW-values. To permit a bias-free evaluation at least eight weeks elapsed between the first (standard approach) and the second (score-based approach) reading of the bedside CXRs, which were randomly presented. For the first reading, radiologists were asked to classify the bedside CXRs as showing the presence or absence of a pulmonary edema. Prior to the score-based evaluation, the radiologists were introduced to the application for approximately five minutes. For the reading where the software had been altered, the radiologists were not informed of the software score, nor did they know the scores of the various indications or the threshold distinguishing between pulmonary edema and no pulmonary edema. Details of the readers’ evaluation results for both reads (pulmonary edema yes/no/ system’s score), the time required to assess the bedside CXR with and without the score-based system as well as any software related problems were recorded by a research assistant. Examples of bedside chest radiographs with and without indications of pulmonary edema are shown in Figures 2 and 3.

### Statistical methods

Statistical testing was performed using SPSS software (version 19.0, Chicago, Ill, USA) and R software (R Core Team (2014). R: A language and environment for statistical computing. R Foundation for Statistical Computing, Vienna, Austria. URL http://www.R-project.org) with Hmisc package (Frank E Harrell Jr, with contributions from Charles Dupont and many others. (2014). Hmisc: Harrell Miscellaneous. R package version 3.14-4. URL http://CRAN.R-project.org/package=Hmisc). Results were expressed as mean values ± standard deviation. Mean sensitivity, specificity, positive and negative predictive value as well as the percentage of overall agreement was calculated for both the standard and the standardized software-based approach. Confidence intervals were calculated. To show interreader variability, the free-marginal multirater kappa value was calculated for both the standard and the standardized software-based approach. Free-marginal multirater kappa analysis was used as readers were not obliged to assign a certain number of cases to each category. To measure the improvement in prediction performance gained by the proposed standardized scoring software the net reclassification index was calculated for each reader as well as for all readers. The level of statistical significance chosen was p < 0.05.

## Results

### Patient characteristics

Ten patients representing an EVLW-value of ≤ 8 mL/kg (range 4 to 8 mL/kg; = no pulmonary edema; six men, four women, mean age 68.9 ± 16.5 years, range 42–87 years) and ten patients representing an EVLW-value of ≥ 15 mL/kg (range 15 to 21 mL/kg; = presence of pulmonary edema; five men, five women, mean age 67.9 ± 12.6 years, range 49–86 years) were selected for this study. Patients did not present direct lung injury and were not treated with diuretics or dobutamine during CXR imaging or the PiCCO (EVLW) measurements.

### Image interpretation

Seven radiologists evaluated the patients’ bedside CXRs under routine clinical conditions. They determined that all bedside CXRs were of sufficient quality to be interpreted. No data were excluded. No problems appeared during usage of the score-based system.

The score-based evaluation of the radiographs to establish whether pulmonary edema were present took 23 ± 12 seconds and, thus, significantly longer than the standard approach (7 ± 3 seconds). With the standard approach 5 - 8/10 patients (mean: 5.7 ± 1.1) with pulmonary edema and 6 - 10/10 patients (mean: 9.0 ± 1.4) without pulmonary edema were correctly diagnosed. With the score-based evaluation 6 - 9/10 patients with pulmonary edema (mean: 7.7 ± 1.0) and 10/10 patients without pulmonary edema were correctly diagnosed. The score-based evaluation improved the radiologists’ mean sensitivity to correctly assess pulmonary edema from 57 to 77% as well as their mean specificity from 90 to 100%. The positive predictive value (PPV) was raised from 85 to 100% and the negative predictive value (NPV) from 68 to 81%. A net reclassification index of 0.3 (all readers; p < 0.01; confidence interval: 0.18 – 0.42) demonstrates an improvement in prediction performance gained by the score-based approach. The free-marginal multirater kappa value increased from 0.34 to 0.68 with the use of the standardized software-based approach. The percentage of overall agreement was 67% with the standard approach and 84% with the software-based approach. Detailed information is given in Table 2.

**Table 2:** **Performance of the readers with the score-based and with the standard approach**

	Score-based approach		Standard approach		
	Pulmonary edema	No pulmonary edema	Pulmonary edema	No pulmonary edema	Net reclassification index
Reader 1 (3)	8	10	5	10	0.3 (0.04; 95% CI: 0.02 – 0.58)
Reader 2 (3)	6	10	5	10	0.1 (0.56; 95% CI: -0.23 – 0.43)
Reader 3 (3)	8	10	6	6	0.6 (0.003; 95% CI: 0.21 – 0.99)
Reader 4 (4)	8	10	5	9	0.4 (0.02; 95% CI: 0.06 – 0.74)
Reader 5 (4)	7	10	5	9	0.3 (0.06; 95% CI: -0.01 – 0.61)
Reader 6 (6)	9	10	8	10	0.1 (0.29; 95% CI: -0.09 – 0.29)
Reader 7 (7)	8	10	6	9	0.3 (0.06; 95% CI: -0.01 – 0.61)
All readers	54/70	70/70	40/70	63/70	0.3 (0.000002; CI: 0.18 – 0.42)
Mean sensitivity [%]	77.14 (95% CI: 65.28 – 85.99)		57.14 (95% CI: 44.78 – 68.72)		
Mean specificity [%]	100 (95% CI: 93.52 -100)		90.00 (95% CI: 79.90 – 95.55)		
Positive predictive value [%]	100 (95% CI: 91.73 – 100)		85.11 (95% CI: 71.08 – 93.31)		
Negative predictive value [%]	81.40 (95% CI: 71.25 – 88.67)		67.74 (95% CI: 57.15 – 76.85)		
Free-marginal multirater kappa value	0.68		0.34		
Percentage of overall agreement [%]	67		84		
Duration [seconds]	23 ± 12		7 ± 3		

The first seven rows show the numbers of correct diagnoses (10 bedside CXRs total) during the assessment of ten bedside chest radiographs with and without pulmonary edema each, practicing the score-based and the standard approach (extravascular lung water (EVLW) measurements determined by a pulse contour cardiac output (PiCCO) system served as reference standard). Work experience of the participating radiologists in years is shown in brackets. The net reclassification index demonstrates the improvement in prediction performance gained by the score-based approach (p-values and confidence intervals are shown in brackets). Sensitivities, specificities, positive and negative predictive values, free-marginal multirater kappa values, the percentage of overall agreement and duration are shown for both approaches. The confidence level is 95%. CI = Confidence interval.

## Discussion

The results of this study show that a score-based approach improves radiologists’ diagnostic accuracy during the assessment of pulmonary edema on bedside CXRs (sensitivity: 77 vs. 57%, specificity: 100 vs. 90%, PPV: 100 vs. 85%, NPV: 81 vs. 68%). Further, the proposed approach improved the interreader agreement (free-marginal multirater kappa value: 0.68 vs. 0.34) and the prediction performance (net reclassification index: 0.3). The score-based system allows a standardized analysis of characteristic indications of pulmonary edema and facilitates a structured and objective evaluation.

Especially critically ill patients can potentially benefit from this improved evaluation of their current fluid balance as the hemodynamic status is one of the most often adjusted care variables. Although there are several more sensitive methods of monitoring a patient’s hemodynamic status, it is crucial that as much accurate information as possible be obtained from bedside CXRs since this examination is commonly used in clinics on critically ill patients [30].

Interestingly, no additional benefit was observed with patients undergoing mechanical ventilation in an ICU when CXR was performed daily compared to when only clinically indicated CRXs were performed [31]. This is presumably caused by the limited validity of the evaluation of the hemodynamic status and the presence of pulmonary edema. Nevertheless, it has been shown that 22% of all routine CXRs and 40% of non-routine CXRs led to a change in the treatment of patients in an ICU [32]. In selected cases more reliable, invasive methods are used to assess the hemodynamic status, since these methods are costly and associated with safety concerns [33, 34]. Therefore, simple, non-invasive diagnostic methods, such as the bedside radiograph, are still essential under normal clinical conditions and maximum exploitation of such methods should be aimed for. This may well lead to a reduction in the number of invasive procedure-related adverse events and has the potential of improving the treatment of critically ill patients. Additionally, the CXR evaluation provides a significant increase in important information about the patient’s condition, such as the presence of an infiltrate or a pneumothorax or about the position of artificial devices such as central venous catheters or the endotracheal tube [35]. There is no comparable, commonly available and cost-effective diagnostic approach which offers such a wide spectrum of information as that provided by bedside CXRs [36, 37].

When using the score-based system the radiologist took about 15 seconds longer to interpret each bedside CXR. A newly implemented approach naturally takes longer than a familiar routine. It can be assumed, however, that over time this difference will decrease. Nevertheless, in the context of the trend of an increasing image load [38], investing additional time for each beside CXR can present a challenge. However, in the case of critically ill patients, where the reader’s decision has an important impact on deciding the relevant treatment of the patient and its result [32], time and financial considerations should not be the deciding factors. Further, a tool which provides a more accurate evaluation of bedside CXRs has the potential to reduce the patient’s treatment costs by reducing the need for costlier methods. In order to increase the acceptance for the proposed scoring system it has been implemented as a freely available, user-friendly web-based application.

Our study faces some limitations which suggest directions for future work. There are controversial discussions about the EVLW-value in that it groups patients according to the presence of pulmonary edema [8, 19, 22–26]. Therefore, we established a threshold which is generally accepted as reliably distinguishing patients with and without pulmonary edema and which ignores borderline cases (EVLW-value between 9 and 14 mL/kg). As a consequence, in this study each patient’s lung could be assessed as being hypervolemic or non-hypervolemic. In the case of score-based assessment we established a threshold which distinguishes between hypervolemic and non-hypervolemic patients. When assessing bedside CXRs for pulmonary edema, the radiologist should make a clear (yes/no) decision. Since the EVLW is a ratio scale, further studies need to be done to evaluate a possible correlation between the EVLW-value and the result determined by the scoring system without excluding borderline cases.

Due to the fact that we only assessed 10 bedside CXRs showing pulmonary edema we are not able to establish which imaging criteria best correlate with the EVLW categories. However, Table 1 shows weighting of the different criteria based on findings of previously published research [1, 13, 28, 29]. A higher maximum score means that the corresponding criterion shows extravascular water more distinctively. For example, the criterion “enlarged hilar vessels” got a maximum score of 3 points because it may have multiple causes and it does not show extravascular water indeed. In contrast, the criteria “extensive perihilar haze” and “diffuse increase in density” are caused by and actually represent extravascular water and therefore got a maximum score of 15 points.

Further, we did not distinguish between the different causes of pulmonary edema. Several authors determined criteria to differentiate between cardiogenic, renal, and injury patterns of pulmonary edema with the CRXs being performed predominantly with the patient in an upright position and with a posterior-anterior radiation beam [13, 15, 39]. However, we included bedside CXRs with an anterior-posterior radiation beam and the objective of the present study was to assess pulmonary edema independently of its cause.

## Conclusions

As suggested by our data, the usage of a score-based system which allows a standardized assessment of a patient’s hemodynamic status as shown on bedside CXRs improves the diagnostic accuracy of radiologists, raises the degree of interreader agreement and improves the prediction performance. This is extremely valuable because information acquired through the evaluation of bedside CXRs may well have an instant impact on the treatment of a patient. Further research is required into the performance of the scoring system in the case of borderline EVLW-values. Besides, it would be interesting to investigate if the score detects EVLW alterations.

Our score-based system is available as a charge-free web-based application (accessible via: http://www.radiologie.uk-erlangen.de/aerzte-und-zuweiser/edema).

### Key points

- *Evaluation of a score-based system which allows standardized pulmonary edema assessment of bedside chest radiographs (CXRs).*
- *Extravascular lung water (EVLW) values were determined instantly after bedside CXR imaging and served as a reference standard.*
- *Score-based assessment improved radiologists’ sensitivity from 57 to 77%, specificity from 90 to 100% and the free-marginal multirater kappa value from 0.34 to 0.68. The positive predictive value was raised from 85 to 100% and the negative predictive value from 68 to 81%. The percentage of overall agreement was raised from 67 to 84% and the prediction performance was improved by 30%.*
- *The score-based system is available as a charge-free web-based application.*

## Abbreviations

PiCCO: Pulse contour cardiac output
EVLW: Extravascular lung water
ICU: Intensive care unit
CXR: Chest radiograph.
